# Adherence to subcutaneous interferon beta-1a treatment among patients with relapsing multiple sclerosis: the MAIN-MS study

**DOI:** 10.3389/fneur.2023.1257455

**Published:** 2023-11-28

**Authors:** Raed Al-Roughani, Magd Zakaria, Edward J. Cupler, Karim Taha

**Affiliations:** ^1^Division of Neurology, Amiri Hospital, Sharq, Kuwait; ^2^Department of Neurology, Ain Shams University, Cairo, Egypt; ^3^Division of Neurology, Department of Neurosciences, King Faisal Specialist Hospital and Research Centre, Riyadh, Saudi Arabia; ^4^Neurology and Immunology Medical Affairs, EMEA Region, Merck Serono Middle East FZ-Ltd., Dubai, United Arab Emirates

**Keywords:** adherence, relapsing multiple sclerosis, subcutaneous interferon beta-1a, patient support programme, Rebif

## Abstract

**Introduction and background:**

Adherence is a critical factor for optimal clinical outcomes in multiple sclerosis (MS) treatment. This study investigated the adherence and clinical outcomes of MS patients treated with subcutaneous (sc) interferon (IFN) (β)-1a, an established immunomodulatory treatment for relapsing MS. The benefits of a patient support programme (PSP) were also studied.

**Methods:**

This phase-IV prospective, observational multicentre study enrolled patients with relapsing MS who were treated with sc IFN β-1a for 24 months was conducted at 53 centres across 17 countries. The primary endpoint was adherence to sc IFN β-1a treatment, as assessed using Morisky Green Levine Medication Adherence Scale (MGLS) scores at 24 months. The MGLS is a self-reported diagnostic tool to address medication non-adherence, with a score ranging from 0 to 4, with 0 representing high adherence, 1–2 representing medium adherence, and 3–4 representing low adherence. Other endpoints included time to study and treatment discontinuation over 24 months, the proportion of relapse-free patients, and Expanded Disability Status Scale (EDSS) progression (defined as ≥1.0 point increase sustained for 3 months) at 24 months. A subgroup analysis was performed for endpoints based on patients assigned to PSP (yes/no—PSP versus non-PSP subgroup).

**Results:**

Of the 577 patients enrolled, 408 had evaluable MGLS scores at 24 months. A total of 336 (58.2%; 95% confidence interval [CI]: 54.1–62.3%) patients reported high adherence, 57 (9.9%; 95% CIs: 7.6–12.7%) reported medium adherence, and 15 (2.6%; 95% CI: 1.5–4.3%) reported low adherence at 24 months. The PSP subgroup reported higher adherence (n = 206; 65.8%) than the non-PSP subgroup (n = 130; 56.5%). By 24 months, 52.2% of the patients were relapse-free and 17.2% patients experienced ≥1 relapse. Expanded Disability Status Scale progression was observed in 12.3% of patients. Over the 24-month period, 30.8% of the patients discontinued treatment, and the most common reasons for treatment discontinuation were adverse events (AEs, 10.4%), being lost to followup (7.1%), and a lack of efficacy (5.5%). Overall, 39.6% patients experienced ≥1 AE, which ranged from mild to moderate.

**Conclusion:**

The study demonstrated high adherence to sc IFN β-1a treatment with an added benefit of PSP participation. More than half of the patients remained relapse-free over a 24-month period. No new safety concerns to sc IFN β-1a treatment were observed.

**Clinical trial registration:**

https://clinicaltrials.gov/study/NCT02921035, NCT02921035.

## 1 Introduction

Multiple sclerosis (MS) is an inflammatory, demyelinating, neurodegenerative, and progressive autoimmune disease of the central nervous system (1). The exact aetiology of MS is not known. Various hypotheses have linked infection, genetics, and a possible autoimmune mechanism to MS (2). In most cases, patients with MS first experience an acute inflammatory demyelinating event and progress to relapsing–remitting MS, which is marked by distinct acute events of deteriorating neurological function (relapses) followed by partial or total recovery (remission). The relapsing*–*remitting disease course may evolve into a secondary progressive stage with or without occasional relapse, minor remissions, and plateaus (3). MS imposes a considerable burden on patients, caregivers, and society because of the early age of MS disease onset (4). Without a definitive cure for MS, disease-modifying drugs (DMDs) are the current mainstream treatment, aiming to alleviate symptoms, reduce the number of MS relapses, prevent new lesion formation, and potentially slow down disability progression (5–7).

As MS is a chronic condition, its successful treatment requires adherence to prolonged therapy. Maintaining a dosing regimen is critical for achieving the defined therapeutic outcomes. Several studies have shown that adherence to MS treatment is associated with fewer relapses, a slower rate of disease progression, less patient disability, reduced healthcare resource utilisation, lower costs, and improved quality of life (8, 9). However, evidence suggests that adherence to MS treatments is suboptimal (10), with 41–88% of patients not adhering to the DMDs as prescribed (11). In addition, poor drug adherence or treatment gaps have been linked to a higher risk of relapse (12).

The treatment gap associated with DMD use is frequently influenced by patient beliefs and choices, resulting in an unmet need for effective long-term treatment support and management. Several strategies have been investigated and implemented to improve drug adherence, including patient support programmes (PSPs) and innovative injectable devices (13, 14). PSPs may improve treatment adherence as well as clinical outcomes (13, 15). Real-world adherence studies on RebiSmart®, an autoinjector device for MS treatment, have shown that the device helps patients adhere to therapy and potentially improve clinical outcomes (14, 16).

Subcutaneous interferon beta-1a (sc IFN β-1a) is a well-established immunomodulating DMD for clinically isolated syndrome and relapsing multiple sclerosis (RMS) (17) with an estimated cumulative exposure of 1,908,836 patient-years (until September 2022) (18). The effectiveness of sc IFN β-1a in lowering MS disease activity (as measured by clinical outcomes and magnetic resonance imaging surrogate measures) has previously been demonstrated (17, 19, 20). In clinical trials, 22 or 44 μg three times weekly (tiw) sc IFN β-1a has been shown to have a positive benefit, to risk profile and is well tolerated (17, 19, 20). In addition to sc IFN β-1a, the current landscape of MS therapies includes injectables such as other interferons (intramuscular IFN β-1a and IFN β-1b) and glatiramer acetate; oral DMDs such as teriflunomide, dimethyl fumarate, fingolimod, cladribine tablets, and siponimod; and infusion therapies such as natalizumab, alemtuzumab, and ocrelizumab (21–24). Considering patient characteristics, disease severity, the number of alternative therapies (oral and injectable) available for MS management, recent guidelines suggest individualised therapies (21). In the context of an ever-evolving treatment landscape, sc IFN β-1a, recognised for its clinical efficacy and established safety profile, continues to address the unique needs of patients with MS (24).

This study assessed adherence and clinical outcomes of patients with relapsing MS who were prescribed sc IFN β-1a (with or without RebiSmart® device) over a period of 24 months. We also assessed the benefits of additional support through enrolment in a PSP.

## 2 Materials and methods

### 2.1 Study design and population

This was a 24-month, **M**ulticentre, phase-IV, prospective, observational, open-label, single-arm non-interventional study to assess **A**dherence to treatment for pa**TIENT**s with relapsing MS who were prescribed sc IFN β-1a (MAIN-MS). The MAIN-MS study was conducted at 53 centres in 17 countries between June 2016 and July 2020. The study population comprised patients with relapsing MS who were either treatment naïve or treated with other DMDs and who were prescribed sc IFN β-1a treatment.

### 2.2 Eligibility

Patients were eligible if they fulfilled the following inclusion criteria: (1) aged ≥18 years and ≤ 60 years at the time of sc IFN β-1a initiation; (2) diagnosed with relapsing MS according to the 2017 McDonald criteria (25); (3) treatment naïve or receiving other DMDs but would switch to sc IFN β-1a, a human serum albumin-free formulation (with or without RebiSmart® device), at a dosage of 44 μg, tiw; and (4) provided signed informed consent. All the enrolled patients were followed up for 24 months with no additional visits or intervention(s) outside of the investigators’ routine practice. Patients could also be assigned to a PSP at the discretion of the investigator. Patients who withdrew from the study prematurely were not replaced.

### 2.3 Study sample size

Assuming a 5% dropout rate, 526 patients were planned to be enrolled in the study to provide a sample size of 500 evaluable patients. With a precision of at least 4.4% using 95% confidence intervals (CIs), the proportion of patients with high Morisky Green Levine Medication Adherence Scale (MGLS) score at 24 months was estimated to be 55%.

### 2.4 Study endpoints

The primary endpoint was adherence to sc IFN β-1a treatment as determined using the MGLS score at 24 months. MGLS is a self-reported diagnostic tool to address medication non-adherence, with scores ranging from 0 to 4, with 0 representing high adherence, 1–2 representing medium adherence, and 3–4 representing low adherence (26). Other endpoints included time to study and treatment discontinuation over 24 months, reasons for study and treatment discontinuation, the proportion of relapse-free patients at 24 months, and the proportion of patients with Expanded Disability Status Scale (EDSS) progression at 24 months. EDSS progression was defined as an EDSS score increase of ≥1.0 point that was sustained for at least 3 months at 24 months. The safety profile of sc IFN β-1a, including adverse events (AEs), serious AEs (SAEs), and adverse drug reactions, was also assessed.

### 2.5 Statistical analyses

Descriptive statistics were used for all continuous variables. The 95% CIs were provided wherever applicable. The full analysis set (FAS) was defined as all patients who provided informed consent and met the eligibility criteria. Baseline and efficacy data were analysed on the basis of FAS. The safety analysis set (SAF) was defined as all patients who provided informed consent and received at least one dose of study treatment. Safety was analysed using the SAF. Subgroup analysis was planned for endpoints based on (1) patients assigned to PSP (yes/no—PSP versus non-PSP subgroup and (2) patients assigned to the RebiSmart® device (yes/no: RebiSmart® versus non-RebiSmart® subgroup).

Patients who discontinued the study were categorised under study discontinuation, and patients who discontinued sc IFN β-1a therapy were categorised under treatment discontinuation. The time to study discontinuation over the 24-month period and the time to treatment discontinuation over the 24-month period were summarised using descriptive statistics and the Kaplan–Meier estimate of probability.

The safety evaluation included the summary of AEs and SAEs during the study period. All AEs were coded using the Medical Dictionary for Regulatory Activities (MedDRA) version 23.0 terminology for the System Organ Class and Preferred Term.

Statistical analysis system (SAS) version 9.1.3 or higher was used to perform statistical analysis. The overall MGLS score and each of the adherence categories (high, medium, or low) were summarised as numbers and percentages from FAS at 24 months or early withdrawal, with 95% CIs provided for each adherence category. The last observation carried forward (LOCF) method was used to perform sensitivity analysis of the primary endpoint.

### 2.6 Ethics statement

Ethical approval (MOH-2021-1670) was obtained from the institutional review boards at the Ain Shams University and at each of the other participating centres of the respective countries in accordance with their ethical regulations and was conducted according to the Declaration of Helsinki. An informed consent form was signed by each patient. The patients were free to withdraw consent at any time without prejudice to their medical care and were not obliged to state their reasons.

### 2.7 Data availability statement

The datasets generated during and/or analysed during the current study are available from the corresponding author on reasonable request.

In addition, any requests for data by qualified scientific and medical researchers for legitimate research purposes will be subject to Merck Data Sharing Policy. All requests should be submitted in writing to Merck’s data sharing portal: https://www.merckgroup.com/en/research/our-approach-to-research-and-development/healthcare/clinical-trials/commitment-responsible-data-sharing.html. When Merck has a co-research, co-development, or co-marketing or co-promotion agreement, or when the product has been outlicensed, the responsibility for disclosure might be dependent on the agreement between parties. Under these circumstances, Merck will endeavour to gain agreement to share data in response to requests.

## 3 Results

### 3.1 Patient disposition

A total of 584 patients were screened, of which 577 were enrolled in the study and constituted the FAS. Of the 577 enrolled patients, 94.1% (n = 543) initiated treatment with sc IFN β-1a and were included in the SAF. Overall, 36.2% (n = 209) of the patients discontinued the study.

### 3.2 Patient demographics and baseline disease characteristics

The majority of the patients were women (69.3%) and had a mean (standard deviation [SD]) age of 34.3 (9.3) years. The baseline demographics and MS disease history are described in Table 1. Amongst the patients included in the FAS, 21.5% (n = 124) used RebiSmart®. A total of 54.2% (*n* = 313) of the patients were assigned to the PSP.

**Table 1:** Baseline demographics and disease characteristics (FAS).

Characteristics	*N* = 577
Mean age, years (SD)^a^	34.3 (9.3)
Female, *n* (%)	400 (69.3)
Mean time since first symptoms of MS, years (SD)	4.6 (5.3)
Median time since first symptoms of MS, years (range)	2.7 (0–33.8)
Mean time since first relapse, years (SD)	2.6 (3.9)
Median time since first relapse, years (range)	0.9 (0–28.8)
Mean number of relapses in the previous 24 months (SD)	1.2 (0.9)
Median number of relapses in the previous 24 months (range)	1 (0–6)
Classification, *n* (%)	
McDonald criteria	425 (73.7)
Clinically definite	152 (26.3)
Mean EDSS score (SD)^b^	1.8 (1.1)
Median EDSS score (range)^b^	1.5 (0–6)

a Two outlier patients aged 17.3 and 61.5 years were included in the FAS and SAF.

b *n* = 576.

EDSS, Expanded Disability Status Scale; FAS, full analysis set; MS, multiple sclerosis; n, number of patients; SD, standard deviation.

### 3.3 Adherence

The adherence to sc IFN β-1a was assessed at 24 months. Overall, 70.7% (n = 408) of the patients had evaluable MGLS scores at 24 months. The majority (n = 336) of the patients reported high adherence to sc IFN β-1a (58.2% of 577 patients; 95% CI: 54.1–62.3%). Fifty-seven patients (9.9%; 95% CI: 7.6–12.7%) reported medium adherence, whereas only a few patients reported low adherence (2.6%; 95% CI: 1.5–4.3%) (Table 2). However, reports on adherence were missing for 135 (23.4%; 95% CI: 20.1–27.2%) patients and not evaluable for the remaining 34 (5.9%; 95% CI: 4.1–8.2%) patients.

**Table 2:** Adherence at 24 months (FAS).

	All patients (*n* = 577)	95% CI
MGLS score, *n* (%)		
0	336 (58.2)	
1	36 (6.2)	
2	21 (3.6)	
3	5 (0.9)	
4	10 (1.7)	
Missing	135 (23.4)	
Not evaluable^a^	34 (5.9)	
Adherence, *n* (%)		
High	336 (58.2)	(54.1–62.3)
Medium	57 (9.9)	(7.6–12.7)
Low	15 (2.6)	(1.5–4.3)
Missing	135 (23.4)	
Not evaluable^a^	34 (5.9)	

A MGLS score of 0 indicates high adherence; a score of 1–2 indicates medium adherence; a score of 3–4 indicates low adherence.

a Patients who did not take at least one dose of study medication.

CI, confidence interval; FAS, full analysis set; MGLS, Morisky Green Levine Medication Adherence Scale; n, number of patients.

The sensitivity analysis using the LOCF method performed to include patients who had a missing MGLS score at 24 months or had an early withdrawal (23.4%) resulted in data availability for all 543 patients who initiated treatment. Including these data, the proportion of patients with high adherence was 76.8% (95% CI: 73.1–80.2%).

The last available MGLS assessments revealed that 107 (79.3%) patients had high adherence, 20 (14.8%) had medium adherence, and 8 (5.9%) had low adherence.

**Figure 1:**
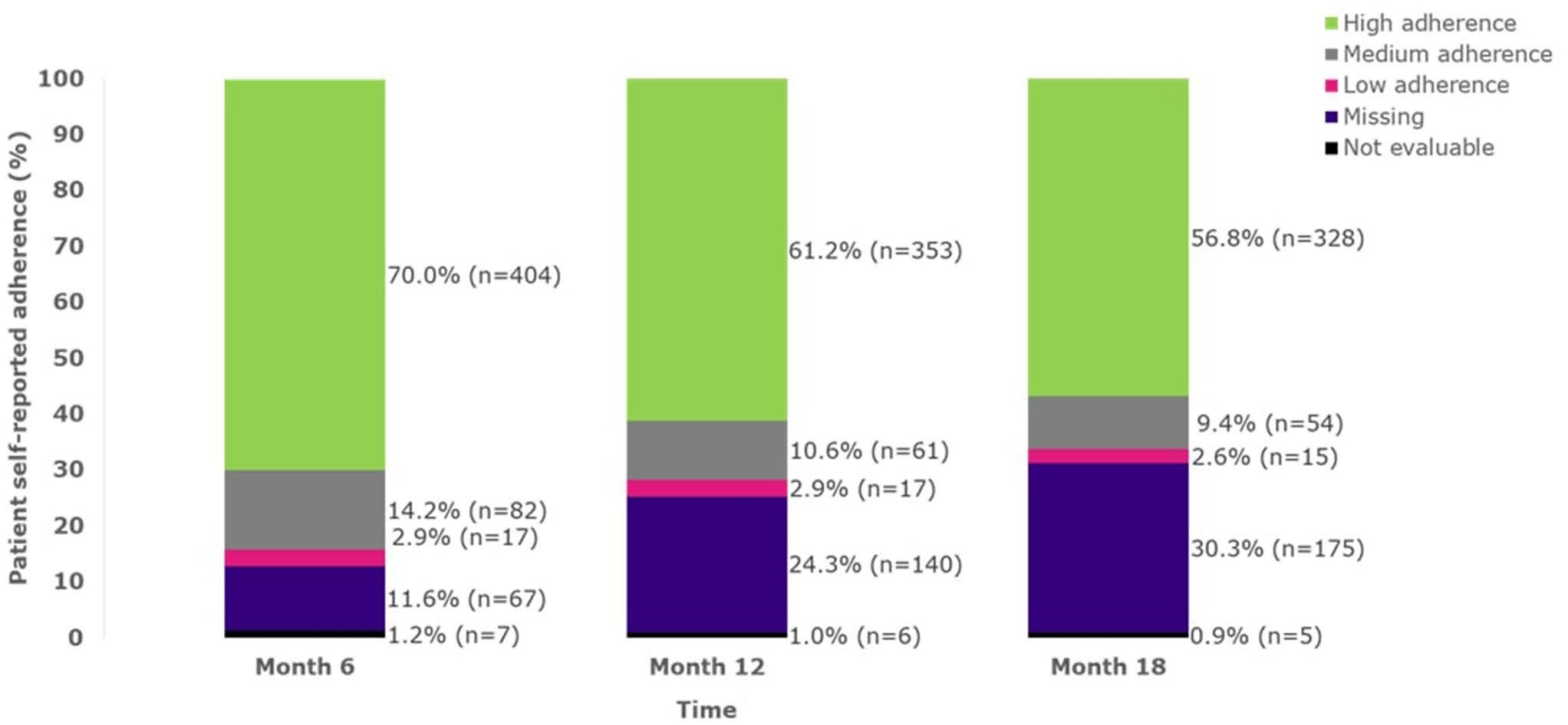
Adherence over time up to 18 months (FAS). A MGLS score of 0 indicates high adherence; a score of 1–2 indicates medium adherence; a score of 3–4 indicates low adherence. FAS, full analysis set; MGLS, Morisky Green Levine Medication Adherence Scale; n, number of patients.

Adherence to sc IFN β-1a remained relatively stable over the study period (Figure 1) when considering evaluable non-missing reports. Patients with evaluable non-missing MGLS scores at pre-specified timepoints continued to report high adherence (Figure 1).

### 3.4 Study discontinuation

**Figure 2:**
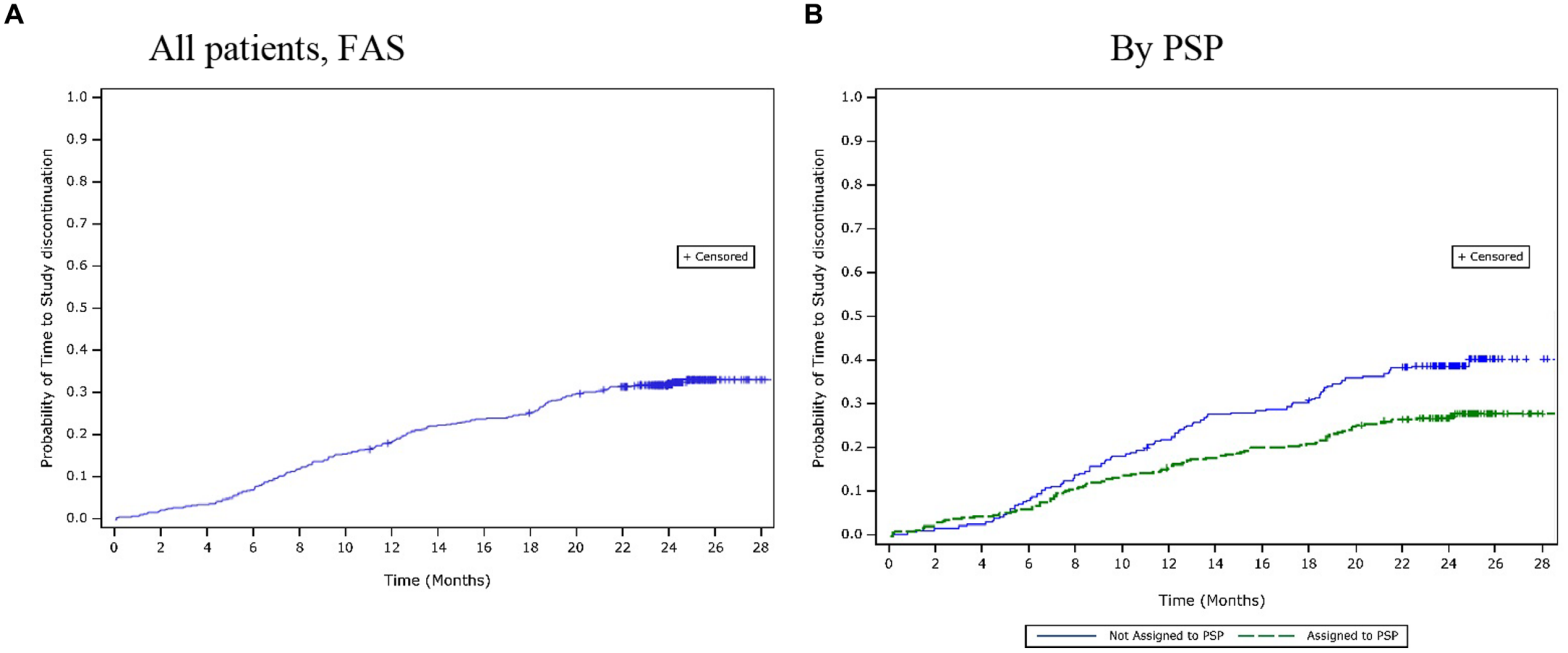
Time to study discontinuation over 24 months. **(A)** All patients, (FAS). **(B)** By PSP. FAS, full analysis set; PSP, patient support programme.

Over the 24-month period, 36.2% (*n* = 209) of patients discontinued the study. The mean (SD) time to study discontinuation was 10.1 (7.1) months. Based on the Kaplan–Meier estimates, the time to study discontinuation for the first 25% of patients was 13.4 months (Figure 2A). The most common reasons for study discontinuation were AEs (10.1%), patients lost to follow-up (6.9%), and a lack of efficacy (5.7%). The time to study discontinuation was observed to be similar across the adherence categories, and there were no clear differences in the reasons for study discontinuation amongst different adherence categories.

### 3.5 Treatment discontinuation

**Figure 3:**
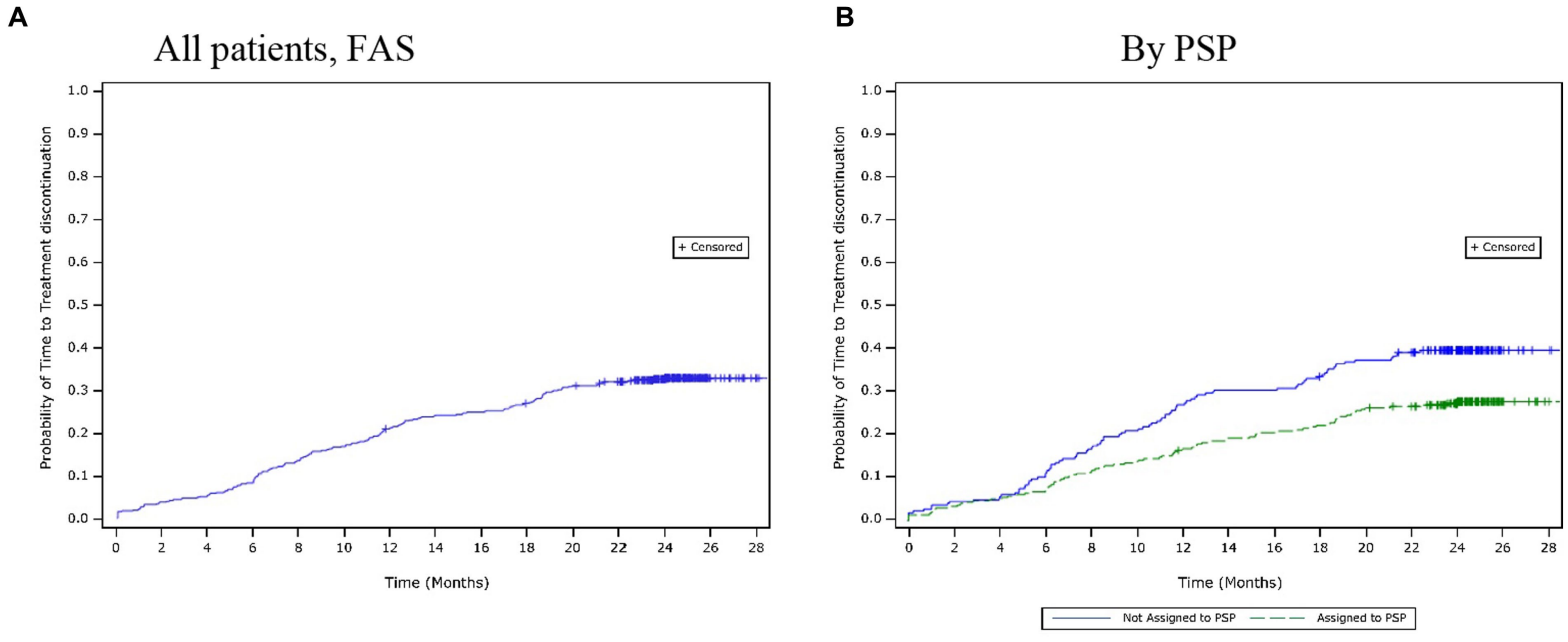
Time to treatment discontinuation over 24 months. **(A)** All patients, (FAS). **(B)** By PSP. FAS, full analysis set; PSP, patient support programme.

Over the 24-month period, 30.8% (n = 178) of patients discontinued their treatment. The mean (SD) time to treatment discontinuation was 10.3 (6.4) months. Based on the Kaplan–Meier estimates, the time to treatment discontinuation for the first 25% of patients was 16.2 months (Figure 3A). The most common reasons for treatment discontinuation were AEs (10.4%), lost to follow-up (7.1%), and a lack of efficacy (5.5%). The time to treatment discontinuation was observed to be similar across the adherence categories, and there were no clear differences in the reasons for treatment discontinuation amongst different adherence categories.

### 3.6 Relapses and EDSS progression

By 24 months or early withdrawal, >50% of the patients were relapse-free (52.2%) and 17.2% of patients experienced at least one relapse. No information was available for the remaining 30.7% of the patients. The mean (SD) number of relapses were 1.1 (0.4), with a maximum of three relapses. The median duration of the relapse was 23.0 days (interquartile range: 12.0–43.0). Amongst the patients who were relapse-free over 24 months, 67.4% reported high adherence, 25.3% reported medium adherence, and 6.4% reported low adherence, whereas amongst the patients with at least one relapse, 60.6% reported high adherence, 32.3% reported medium adherence, and 6.1% reported low adherence.

At 24 months or early withdrawal, EDSS progression was observed in 12.3% (n = 71) of the patients, and most of these patients (n = 63, 10.9%) had an EDSS score of ≤3, whereas 1.2% (n = 7) of the patients had EDSS score of >3. The EDSS score was available for 70.4% (n = 406) of patients at 24 months: 59.6% (n = 344) of patients had an EDSS of ≤3 and 10.7% (n = 62) of patients had an EDSS of >3. Throughout the study, most patients had an EDSS score of ≤3: 87.7% (n = 506) patients at baseline, 75.2% (n = 434) patients at 6 months, 65.7% (n = 379) at 12 months, 60.8% (n = 351) patients at 18 months, and 59.6% (n = 344) patients at 24 months.

### 3.7 Subgroup analysis (PSP versus non-PSP subgroup)

The PSP subgroup (n = 206; 65.8%) had a larger proportion of patients reporting high adherence compared with the non-PSP subgroup (n = 130; 56.5%). A lower proportion of patients had discontinued the study by 24 months in the PSP subgroup (27.8%) than in the non-PSP subgroup (39.6%). The mean (SD) times to study discontinuation were similar between the PSP (11.2 [6.9] months) and the non-PSP (11.5 [6.2] months) subgroups. However, the Kaplan–Meier estimates showed that the estimated time to study discontinuation for the first 25% of patients was longer in the PSP subgroup (20.0 months) than in the non-PSP subgroup (12.7 months; Figure 2B). The most common reasons for study discontinuation were similar between both subgroups. However, a higher proportion of patients in the non-PSP subgroup than in the PSP subgroup reported study discontinuation due to AEs (non-PSP subgroup: 12.6% vs. PSP subgroup: 9.3%), lack of efficacy (non-PSP subgroup: 10.4% vs. PSP subgroup: 2.9%), withdrawal of consent (non-PSP subgroup: 3.9% vs. PSP subgroup: 3.5%), and financial issues (non-PSP subgroup: 3.0% vs. PSP subgroup: 0.0%).

Similar results were reported for treatment discontinuation. A lower proportion of patients had discontinued the treatment by 24 months in the PSP subgroup (27.8%) than in the non-PSP subgroup (39.6%). The mean (SD) time to treatment discontinuation was longer in the PSP subgroup (10.7 [6.9] months) than in the non-PSP subgroup (10.0 [6.0] months). However, the Kaplan–Meier estimates showed that the estimated time to treatment discontinuation for the first 25% of the patients was longer in the PSP subgroup (19.5 months) than in the non-PSP subgroup (11.5 months; Figure 3B). The most common reasons for treatment discontinuation were similar between both subgroups. However, a higher proportion of patients in the non-PSP subgroup than in the PSP subgroup reported treatment discontinuation due to AEs (non-PSP subgroup: 13.5% vs. PSP subgroup: 9.3%), lack of efficacy (non-PSP subgroup: 10.0% vs. PSP subgroup: 2.9%), and financial issues (non-PSP subgroup: 3.0% vs. PSP subgroup: 0.0%).

Compared with the PSP subgroup (16.3%), the non-PSP subgroup (20.4%) had more patients with at least one relapse. The proportions of patients with EDSS progression were comparable between both subgroups.

### 3.8 Safety

The median duration of exposure to sc IFN β-1a over the study period was 729 days. Of the 543 patients who initiated sc IFN β-1a, 39.6% experienced at least one AE, and the AEs were mild to moderate in nature. The most frequent AEs reported for ≥2% of patients were influenza-like illness (7.4%), pyrexia (6.8%), headache (3.1%), MS relapse (3.1%), asthenia (2.9%), and injection site reaction (2.2%).

A total of 5.2% of patients experienced at least one SAE. MS relapse was the most commonly reported SAE, with 15 patients experiencing MS relapses requiring hospitalisation. Amongst the remaining SAEs, four were pregnancy-related, and there was one case each of cholecystitis, liver disease, urinary tract infection, weight gain, gait disturbance, and psoriasis.

Overall, 30.6% of patients experienced at least one AE related to the study medication, including five (0.9%) patients with at least one SAE. The most frequent treatment-related AEs reported for ≥2% of patients were influenza-like illness (7.4%), pyrexia (6.8%), headache (2.2%), asthenia (2.2%), and injection site reaction (2.2%). Of the five treatment-related SAEs, two cases were of depression and one case each was of anaemia, increased liver function test, and reduced white blood cell count.

## 4 Discussion

Because MS is a chronic disease, long-term adherence to treatment can be challenging to achieve desired clinical outcomes. Adherence to medication is a critical factor for patients with MS, as long-term use of the healthcare professionals (HCPs) prescribed DMDs that are usually associated with reduced relapse rates, reduced disease progression, and increased drug efficacy (9, 11, 12, 27). The most common reasons for failure to adherence to treatment are forgetting to take the medication, patient-perceived lack of efficacy, injection anxiety, as well as AEs related to injection site reactions, flu-like symptoms, and fatigue (28). The use of support strategies (15, 29) and treatment maintenance methods (14, 30, 31) are being investigated to overcome poor adherence.

In this study, it was observed that more than 50% of the patients self-reported high adherence to the sc IFN β-1a treatment as assessed using the MGLS score at 24 months. The sensitivity analysis to include the last observed non-missing MGLS scores further increased the proportion of patients reporting high adherence to 77%. Another important observation in this study was that adherence to sc IFN β-1a treatment remained relatively stable over the study period at 6, 12, and 18 months when considering evaluable non-missing MGLS scores. Patients continued to report high adherence over 6–18 months. Adherence to sc IFN β-1a treatment has been studied previously; a narrative review of the literature on treatment adherence to DMDs by Menzin et al. has reported high medication adherence rates, ranging from 58.5 to 77.6% for sc IFN β-1a treatment in patients with relapsing MS (11).

In this study, because a relatively low number of patients used the RebiSmart® device and the results of subgroup analysis were inconclusive, we have not described them further. However, previous studies have reported that the use of RebiSmart® device assists in improving adherence to sc IFN β-1a therapy (16, 30, 31). The results from the recent REBIFLICT study have shown that high adherence (97.9%) to sc IFN β-1a therapy was achieved with the RebiSmart® device over 24 months. Of note, the REBIFLICT study assessed adherence on the assumption that treatment adherence and clinical data were frequently communicated to patients by their attending physicians (30). The READOUT smart study reported a mean quantitative adherence rate of 85.3% over 24 months (31). The results from the SMART study has also shown that patients with relapsing MS using RebiSmart® device to self-inject sc IFN β-1a had excellent adherence at the end of 12 months and had good clinical outcomes. In addition, patients reported that the RebiSmart® device was convenient and easy to use (16).

Clinical efficacy was evaluated in terms of relapse rates and EDSS outcomes. More than 50% of the patients were relapse-free at 24 months or early withdrawal, and 17.2% patients had experienced mostly 1 to 3 relapses. In the pivotal PRISMS study, 45% and 32% of the patients receiving sc IFN β-1a 44 μg were relapse-free at year 1 and year 2, respectively (20). Moreover, in this study, the median EDSS scores (≤3) were stable during the assessments conducted at pre-specified timepoints throughout the study, and the proportion of patients with EDSS progression was relatively low (12.3%). The beneficial effect of sc IFN β-1a on EDSS progression observed in our study was consistent with previous findings (32–34).

Furthermore, in our study, the adherence to sc IFN β-1a was slightly higher in the patients who were relapse-free compared to patients who had at least one relapse. Previous studies have indicated that adherence to sc IFN β-1a treatment is associated with a lower probability of relapse (35, 36). For example, the STAR study by Huppert et al. showed that the proportion of patients with MS with good adherence were relapse-free at 12 months compared to those with fair or poor adherence (77.6% vs. 50.0%; *p* = 0.0107) (37).

There is limited information on the benefits of PSP participation in MS (29). However, PSPs have been found to provide potential benefits in other therapeutic areas such as cardiovascular disease, diabetes, and asthma (38). Structured PSPs with skilled healthcare workers and specialised nurses enable continuous patient education and provide support to patients initiating immunotherapies, which may have an immense impact on health-related outcomes in patients with MS (39). A large retrospective cohort study (N = 3,993) by Tan et al. evaluated the impact of a speciality care management programme amongst patients with MS. The findings suggested that the speciality care management programme was not only associated with improved treatment adherence and persistence but also reduced MS-related hospitalisations and decreased MS-related costs (13). Notably, a large proportion of patients in the study were receiving interferons as MS medication (intramuscular IFN β-1a: 37.3%; sc IFN β-1a: 16.6%; and IFN β-1b: 13.5%) (13). Another study by Lenz and Harms showed that, amongst patients with mild-to-moderate relapsing-remitting MS, adherence to disease-modifying therapies was significantly higher in PSP participants than in the non-PSP participants (92.9% vs. 61.8%; *p* = 0.0197) irrespective of treatment duration (15). Consistent with these previous reports, in the present study, the proportion of patients reporting high adherence to sc IFN β-1a was higher in the patients assigned to PSP (65.8%) compared to those not assigned to PSP (56.5%). In addition, a lower proportion of patients discontinued the treatment by 24 months in the PSP subgroup than in the non-PSP subgroup. Furthermore, the mean time-to-treatment discontinuation was longer in the PSP subgroup than in the non-PSP subgroup. It must be noted that the patients in the study had a baseline mean and median EDSS of 1.8 and 1.5, respectively, representing a mild active disease. These findings highlight the importance of enhancing and promoting strategies (such as PSPs) to manage patient expectations and AEs and educate patients, families, and caregivers about establishing patient–physician relationships and the benefits of participation in PSPs (40).

Study discontinuations and sc IFN β-1a treatment discontinuations were observed in more than 30% of the patients over the 24 months. The most common reasons for these discontinuations (study and treatment discontinuation) were AEs, being lost to follow-up, and a lack of efficacy. In a real-world cohort study by Espin and Munschauer, the discontinuation rates to sc IFN β-1a treatment were 36.9% in year 1 and 49.5% in year 2 (41). In general, previous study reports have shown higher discontinuation rates with sc IFN β-1a treatment (41, 42). The retrospective (43) and prospective observational (37) studies have shown AEs as one of the most common reason to discontinue IFN-β therapy in real-world settings.

In terms of safety, sc IFN β-1a tiw already has a well-established safety profile supported by more than 20 years of clinical and real-world evidence (44), and most AEs associated with sc IFN β-1a are mild to moderate in nature. The most commonly reported AEs with sc IFN β-1a treatment are influenza-like illness, injection site reaction, and headache (45). Overall, no new safety concerns were observed in our study. Consistent with the known safety profile of sc IFN β-1a, the most frequent AEs (reported by ≥2% of patients) in our study were influenza-like illness, pyrexia, headache, MS relapse, asthenia, and injection site reaction. The five treatment-related SAEs, which included two cases of depression and one case each of anaemia, increased liver function test values, and decreased white blood cell count, were also consistent with the known safety profile of sc IFN β-1a used in the approved indication.

Although our study was not designed to, or aimed at, comparing the treatment adherence of injectable therapies and other approved MS DMDs, this is an important aspect, especially considering the evolving MS treatment landscape and shifting prescription trends. For example, data from a recent cross-sectional study showed significant changes in the initiation patterns of DMDs in MS, with patients receiving treatment earlier in their disease course and a shift towards oral therapies from the platform injectable therapies (46). The exact reasons for this shift were not determined but were attributed to factors such as convenience of administration, insurance limitations, and advertising strategies (46). Of note, prescription trends for MS vary across different regions and countries and treatment choice also depends on the availability of DMDs in the region (47). In general, adherence to the DMDs in MS varies widely between 41% and 93% and has been attributed to factors such as patient-specific factors, therapy-specific factors, management, and healthcare system-related factors (11, 48).

Considering this recent shift in prescription towards oral therapies in MS, it is also pertinent to understand the impact of the route of administration on DMD adherence. The rate of adherence to injectable therapies in patients with MS has been reported as being between 41 and 88% (11). A few studies have compared adherence between oral DMDs and injectable DMDs. A recent study by Martinez et al. reported that a change in the route of administration from injectable to oral was associated with increased adherence (49). Two retrospective analyses of short-term claims data in the US observed higher adherence to the treatment in patients initiating an oral DMD (fingolimod) compared to those initiating injectable DMDs (50, 51). Another retrospective analysis of claims data for MS patients in the US, however, observed no difference in adherence between patients initiating oral DMDs and those initiating injectable treatment (10). The study reported suboptimal adherence with both types of treatment (10). Another US claim analysis, by Munsell et al., assessing prescribing patterns for oral and self-injectable DMDs, reported that the route of administration was not a significant predictor of treatment non-adherence (52). A cross-sectional study in Spain reported higher adherence to the injectable compared to the oral therapies attributed mainly to forgetfulness (53). Furthermore, a meta-analysis by Nicholas et al. assessed the rates of adherence and persistence for once-and twice-daily oral DMDs in patients with MS using real-world data (54). The meta-analysis indicated that approximately 20% patients with MS do not adhere to, and approximately 25% discontinue, daily oral DMDs within 1 year of treatment initiation (54). Therefore, with this limited and varied evidence, it is difficult to establish a clear difference in adherence between oral and injectable DMD therapies, and further research on this topic is warranted.

## 5 Limitations

There were a few limitations of this study that should be considered when interpreting the results. Because this non-interventional study was open-label and observational, there was limited control over the outcome assessments as patient monitoring and diagnoses were performed as per standard care. This could have led to potential enrolment and information bias. Regarding enrolment bias, there is a possibility that the demographic characteristics/disease status of patients who participated in this study may differ from those who did not participate. Information bias may result from differences in collected data, such as accuracy/completeness that may misclassify patients in terms of exposures or outcomes. To minimise these study limitation, standardised case report forms, systematic site training and monitoring, and other guidance documentation were used to ensure consistent data collection. However, the relatively higher study and treatment discontinuation (>30% of the enrolled patients) rates may have affected the results. To limit the effect of missing data on MGLS scores, a sensitivity analysis was performed. Furthermore, variability in the treatments received may limit reliability of the interpretations of the results.

## 6 Conclusion

This non-interventional study demonstrated high patient adherence to sc IFN β-1a treatment as assessed using MGLS. PSP participation had an added benefit as the number of patients reporting high adherence was slightly higher in patients assigned to a PSP than in patients not assigned to a PSP. Favourable clinical outcomes in terms of low relapses could be correlated with high drug adherence. Overall, no safety concerns were observed in this study. The safety profile was consistent with the known safety profile of sc IFN β-1a used for the approved indication. The findings of our study add to the available data regarding treatment adherence with sc IFN β-1a in patients with relapsing MS and may help HCPs to make informed decisions about treatment maintenance.

## Data availability statement

The raw data supporting the conclusions of this article will be made available by the authors, without undue reservation.

## Ethics statement

Ethical approval (MOH-2021-1670) was obtained from the institutional review boards at the Ain Shams University and at each of the other participating centers of the respective countries in accordance with their ethical regulations and was conducted according to the Declaration of Helsinki. An informed consent form was signed by each patient. The patients were free to withdraw consent at any time without prejudice to their medical care and were not obliged to state their reasons. The studies were conducted in accordance with the local legislation and institutional requirements. The participants provided their written informed consent to participate in this study.

## Author contributions

RA-R: Conceptualization, Data curation, Formal analysis, Methodology, Supervision, Writing – review & editing. MZ: Conceptualization, Data curation, Formal analysis, Methodology, Supervision, Writing – review & editing. EC: Conceptualization, Data curation, Formal analysis, Methodology, Supervision, Writing – review & editing. KT: Conceptualization, Data curation, Formal analysis, Methodology, Supervision, Writing – review & editing.
